# Improved Sepsis Alert With a Telephone Call From the Clinical Microbiology Laboratory: A Clinical Trial

**DOI:** 10.1097/MD.0000000000001454

**Published:** 2015-10-02

**Authors:** Eleonora Bunsow, Marcela González-Del Vecchio, Carlos Sanchez, Patricia Muñoz, Almudena Burillo, Emilio Bouza

**Affiliations:** From the Clinical Microbiology and Infectious Diseases Department, Hospital General Universitario Gregorio Marañón, Madrid, Spain (EB, MG-DV, CS, PM, AB, EB); Microbiology Department, School of Medicine, Universidad Complutense de Madrid, Spain (PM, AB, EB); and Ciber de Enfermedades respiratorias, CibeRes, Palma de Mallorca, Spain (PM, AB, EB).

## Abstract

Early sepsis attention is a standard of care in many institutions and the role of different specialists is well recognized. However, the impact of a telephone call from a specialist in Clinical Microbiology upon blood cultures request has not been assessed to the best of our knowledge.

We performed telephone calls followed by an interview with physicians and nurses in charge of adult patients (> 18 years old) whose blood cultures had just been received in the Microbiology Laboratory in a tertiary hospital. Patients were randomly classified in 2 different groups: group A (telephone call performed) and group B (no telephone call). At the end of the telephonic intervention, recommendations on the use of microbiology and biochemical tests as well as on the management and antibiotic therapy of sepsis were made if required.

We included 300 patients. Of those fulfilling standard criteria of sepsis, 30.3% of the nurses and 50% of the physicians immediately recognized it. Advice to optimize the use of biochemical and microbiological tests was provided in 36% of the cases and to improve antimicrobial therapy in 57.6%. The median number of days of antibiotic use in groups A and B were, respectively, 6 days (IQR: 2–12) vs 9 days (IQR: 4–16) *P* = 0.008 and the median number of prescribed daily doses of antimicrobials (6 [IQR: 3–17] vs 10 [IQR: 5–22] *P* = 0.016) were lower in group A. We estimate a reduction, only in the use of antibiotic, of 1.8 million Euros per year.

A telephone call with management advice, immediately after the arrival of blood cultures in the Microbiology Laboratory improves the recognition of sepsis and the use of diagnostic resources and reduces antimicrobial consumption and expenses.

## INTRODUCTION

Sepsis is one of the major challenges of modern medicine. It is an important sanitary problem with high incidence, morbidity, and mortality that affects the population worldwide.^1–4^ Without early recognition and prompt management, patients can develop worse stages of sepsis and death. On the other hand, an appropriate management of sepsis can alter its course toward a favorable evolution.^5,6^

In the last years, several campaigns and guidelines appeared to help Health Care Professionals (HCPs) in the recognition and management of sepsis.^7–10^ However, they were mainly focused on the development of management protocols for severe sepsis and septic shock in intensive care units (ICU) or emergency departments (ED). Neither of these approaches examined in depth the impact of an alert system of sepsis out of ICU or ED nor the role of the clinical microbiologist in an early alert of sepsis.

To that end, we performed a prospective study based on telephone calls followed by an interview with physicians and nurses in charge of patients whose blood cultures (BCs) had just been received by the Microbiology Laboratory in a tertiary hospital. The aim of the present study was to determine the capacity of HCPs in the identification of sepsis and the impact of a telephone call from a specialist in Clinical Microbiology (CM) in the early recognition of sepsis.

## PATIENTS AND METHODS

### Study Setting

This study was conducted at a large teaching hospital in Spain that has 1550 beds and serves a population of ∼ 715,000 inhabitants.

### Study Design and Patient Selection

We performed a prospective clinical trial study from August to December 2012. We selected patients who had blood cultures drawn and sent to the Microbiology Lab during the morning shift (9 am–3 pm, Monday–Friday) and we randomly by opportunity sampling: patients whose clinical history number ended in odd numbers were assigned to group A and those whose clinical history number ended in even numbers were assigned to group B:

1. Group A (CASES definition): group of patients in which we tried a telephone contact followed by the performance of an interview to physicians and/or nurses in charge of the patients.
2. Group B (CONTROLS definition): group of patients without a telephone attempt and thus without performance of interview to physicians and/or nurses.

The survey was performed by telephone interviews using CM trained in infectious diseases. Direct conversation with the physicians and/or nurses in charge of the patients was conducted for this study. In both groups, if BCs turned positive, an immediate telephone call with the result of the Gram stain was performed. We excluded patients under 18 years of age and also those with recent positive BCs.

### Interview and Information About Sepsis

Interviews were conducted with physicians and nurses in charge of the patients. The following data was collected prospectively by the CM using a standardized questionnaire form: nurses were questioned about the BCs collection, treatment, and vital signs. Physicians, for their part, were questioned about the reason for the extracted BCs, treatment, use of microbiology, and biochemical tests and diagnosis imaging. In both HCPs we asked if they thought their patient had sepsis syndrome at the moment of the BCs extraction. At the end of the interview, the staff in CM issued recommendations to physicians regarding further microbiology and biochemical tests, radiologic imaging as well as antimicrobial therapy of sepsis. Advice was based on a sepsis bundle protocol and a brochure of antimicrobial therapy developed by the Infectious Diseases Commission of the hospital. At the end of the interview, we asked physicians to rate the interview on a scale between 0 and 10 points (0 points was not helpful and time consuming and 10 was very helpful for sepsis management).

In addition, we sent information about sepsis to HCPs that included a checklist for the early recognition of sepsis and a protocol about management and treatment of sepsis developed by the Infectious Diseases commission of the hospital.

### Processing of Samples in the Microbiology Laboratory

Blood cultures were processed using BACTEC 9240 (Becton Dickinson Microbiology Systems, Maryland, DE) with continuous agitation. Microorganisms were identified using standard procedures.^11^

### Data Collection

The following clinical data was recorded in both groups: age, sex, unit of admission, Charlson's comorbidity index, McCabe, and Jackson stage, underlying conditions, origin and source of infection, use of biochemical and microbiological tests when BCs were taken and during 72 h after, results of microbiological tests, duration, type, dose, and route of antimicrobial therapy. In addition, we collected information on the length of stay in the hospital, ICU admission and mortality. Clinical records for both groups were reviewed after discharge from the hospital, death or up to 30 days after extraction of blood cultures. In order to compare HCPs knowledge of sepsis, we collected the criteria for systemic inflammatory response syndrome (SIRS) in group A and classified sepsis according to the standard definition.

### Definitions

#### Sepsis

The most widely used set of definitions was developed by a consensus committee of experts in 1992.^12^ They defined a systemic inflammatory response syndrome (SIRS) as 2 or more of the following: tachycardia, tachypnea, hyperthermia or hypothermia, high or low white blood cell count. Sepsis was the combination of suspected or confirmed infection plus SIRS; severe sepsis is sepsis plus organ dysfunction; and septic shock is severe sepsis plus hypotension not responsive to a fluid challenge.

#### McCabe and Jackson Scale

Classification of the severity of the underlying illness into rapidly fatal (death expected in <1 month-score 3), ultimately fatal disease (death expected within 5 years-score 2), and nonfatal (score 1).^13^

#### Charlson's Comorbidity Index

Is a summary measure of 19 comorbid conditions that are rated from 1 to 6 based on disease severity.^14^ In addition, each decade of age > 40 adds 1 point to the score.^15^

#### Origin and Source of Infection

Infection was considered to be community-acquired if the BCs were taken within the first 48 h of admission, after this was considered nosocomial. Health-care-related infections included: patients who recently underwent invasive procedures, patients with long-term intravenous, devices, patients who received chemotherapy or parental nutrition, patients on hemodialysis, and patients from nursing homes.^16^ The source of bacteremia was considered clinically documented if there were focal signs and symptoms.

#### Number of Prescribed Daily Doses (PDDs) and Antimicrobial Drug Costs in Euros (€)

Defined as the average dose prescribed according to a representative sample of prescriptions and the costs were calculated on the basis of the actual dose administered and the purchase price to the institution by the pharmacy, without inclusion of administration costs.

### Clinical Research Ethics Committee

Our institutional Ethics Review Committee and the Spanish National Drug Agency approved this investigation. No informed consent was required. Confidentiality was strictly maintained. This study has been registered at the Clinical Trials register (http://www.clinicaltrials.gov, identifier: NCT02325258).

### Statistical Analysis

Data was analyzed using IBM SPSS software, version 21.0 (Armonk, NY). Qualitative variables appear with their frequency distribution. Quantitative variables are summarized as median and interquartile range (IQR) for nonnormal distribution. Level of statistical significance was set as *P* < 0.05.

## RESULTS

**FIGURE 1:**
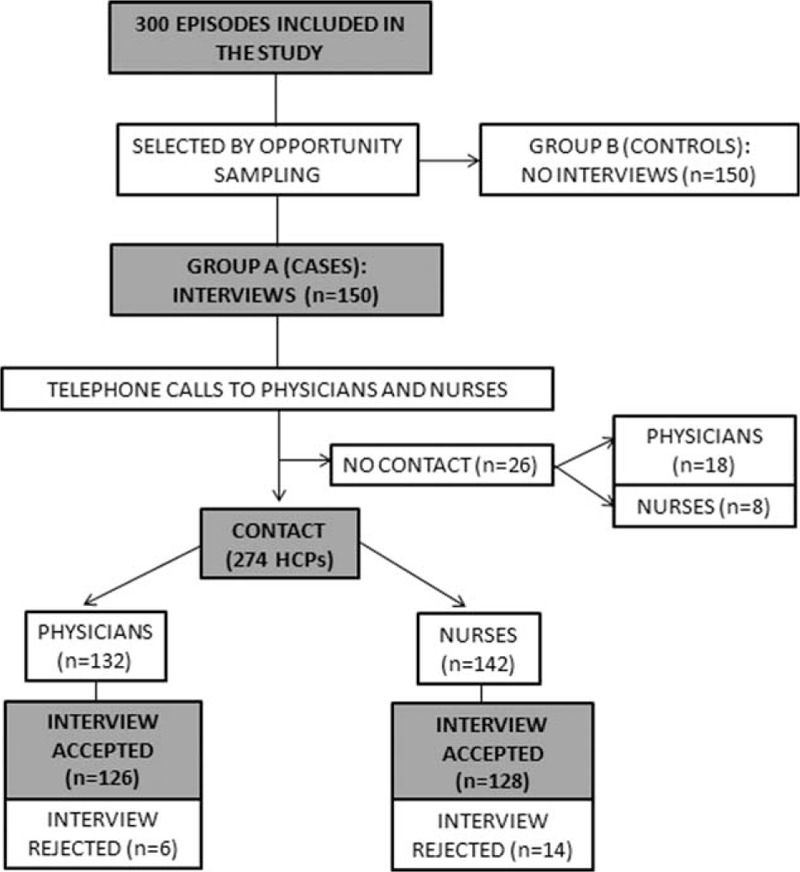
Flow diagram of study patient selection. We enrolled 300 patients with blood cultures drawn that had sent to Microbiology Laboratory. Patients whose clinical history number ended in odd numbers were assigned to group A (intervention) and those whose clinical history number ended in even numbers were assigned to group B (no intervention, control group). We were able to reach telephone contact within 274 HCPs of which, 254 accepted to do the interview (128 nurses and 126 physicians). HCPs = health care professionals.

During the study period, we included 300 patients (150 in group A and 150 in group B). We were able to make telephone contact with 274 (91.3%) professionals (physicians and nurses in group A), of which, 254 (92.7%) accepted to do the interview (128 nurses and 126 physicians). The results of all patients and HCPs included in the study are shown in Figure 1.

### Patient Characteristics

Characteristics of the patients are summarized in Table 1. There were no significant differences in the parameters registered between both groups, before the intervention.

**TABLE 1:**
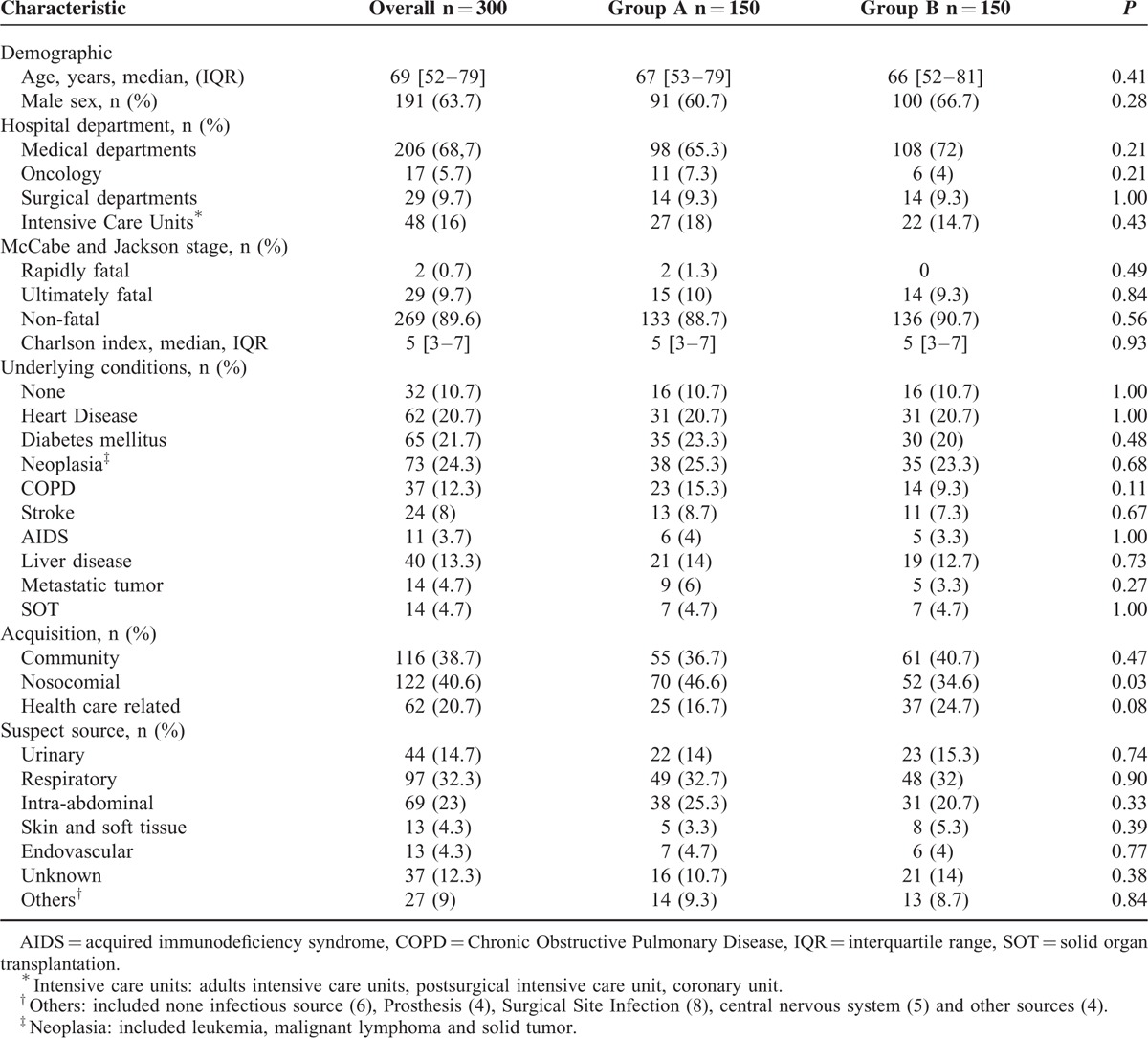
Demographic and Clinical Characteristics of the 300 Patients Included in the Study

### Knowledge and Awareness of Sepsis in Physicians and Nurses

In group A, after a telephone checkup of the sepsis criteria, only 90 patients (60%) fulfilled the definition of sepsis, severe sepsis or septic shock. Of the 90 patients with sepsis criteria, 81.1% were admitted in non-ICU at the time of the interview. The percentage of correct recognition of a case of sepsis by the interviewee was 30.3% by nurses and 50% by the physicians in charge (71.9% in attending physicians and 41.9% in residents). When divided by medical specialties, we found that intensive care physicians recognized a case of sepsis in 100%, emergency department physicians in 73.7%, surgeons in 60%, oncologists in 40%, and other medical specialties in 48.6%.

Of the 60 patients, not fulfilling sepsis criteria, the reasons to obtain BCs were as follows: in 17 (28%) by an automatic order to draw BCs in the presence of fever and without a physician examination of the patient, in 19 (32%) because of focal infection without any signs of SIRS and in 24 (40%) with only one sign of the SIRS definition.

### Use of Resources and Management of Patients with Sepsis Criteria

At the moment of the telephone call, 84.4% of the patients had at least one biochemical test or complete blood count obtained at the time of the BCs; 71.1% had a microbiological test other than blood cultures and 70% had a radiologic imaging obtained. Overall, of all patients fulfilling sepsis criteria, at the time of the interview, 47% did not have a request of lactic acid in blood and 78% did not have a procalcitonin test requested. In total we isolated 56 microorganisms in both groups (16.7% of blood cultures were positive), of which 5% were contaminants. Forty percent of the patients with sepsis criteria were under no antimicrobial therapy at the moment of the telephone interview.

Physicians were satisfied with the interview, with a median rate of 9 on a scale between 0 and 10 points (IQR: 8–10). We did not ask nurses to rate the interview but the general impression of the interviewer was that it was well received.

### Advice Given and Level of Compliance

Of the 125 interviews, advice was given to optimize the use of biochemical and microbiological resources as well as imaging diagnosis in 45 (36%) cases. Their implementation rate was between 35 and 50%. Recommendations to modify antibiotic treatment were given in 48 occasions (57.6%); advice on the management and treatment was highly implemented by the interviewed physicians (78.9%).

We found that, after 72 h BCs were taken, the use of lactic acid was higher in group A vs group B (69 vs 53; *P* = 0.03 respectively). Similar data was found with C-reactive protein (85 in group A vs 71 in group B; *P* = 0.05) and the use of coagulation studies (103 group A vs 88 group B, *P* = 0.02). The use of microbiological tests, other than BCs, was also statistically higher in group A than group B (57.3% vs 42.7%, respectively, *P* = 0.04). The most used alternative microbiological tests were urine (*P* = 0.009) and respiratory sample (*P* = 0.01) cultures.

### Outcome and Expenses

We analyzed outcome in all the patients included in the study. The median of days of antibiotic administration in group A patients was 6 (IQR: 2–12) vs 9 (IQR: 4–16) in group B; *P* = 0.008. Second, the PDDs of antibiotics in group A was 6 (IQR: 3–17.2) and 10 (IQR: 5–22) in group B; *P* = 0.016. The percentage of patients staying in the hospital <1 week after blood culture extraction was 58.1% in group A and 41.9% in group B; *P* = 0.019. We did not find any significant differences in the mortality rate. The differences in patients not admitted to ICU between both groups are shown in Table 2.

**TABLE 2:**
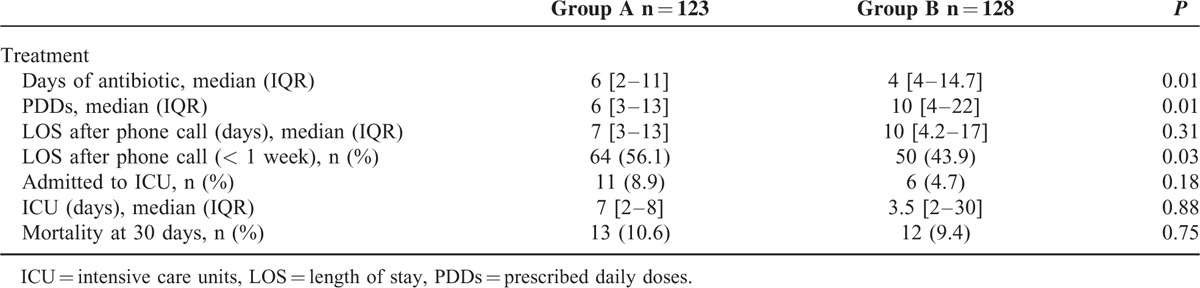
Outcome in All the Patients Included in the Study and Not Admitted to Intensive Care Unit at the Moment of the Blood Cultures Extraction

The median cost in antimicrobial acquisition was 39.8 Euros/patient/day (IQR: 7.67–248.7) in group A (cases) vs 78.5 Euros/patient/day (IQR: 13.08–311.9) in group B (controls), *P* = 0.211. If we could make the phone call to 50% of the blood cultures sent to our Lab and having in mind that the median days with antibiotic per episode was 9, we estimate a reduction, only in the use of antibiotic, of 1.8 million Euros/year.

## DISCUSSION

Our study shows that non-ICU physicians and nurses underestimate the presence of sepsis even in patients to whom they just obtained BCs. A simple interview through a telephone call to physicians and nurses in charge of patients, whose blood cultures have just been received in the Microbiology Laboratory, improves the use of diagnostic resources and reduces antimicrobial consumption.

Sepsis is increasing dramatically,^1,2^ and it is currently one of the most common diseases, exceeding even the incidence of cancer,^17^ HIV/AIDS,^18^ acute myocardial infarction,^19^ and stroke.^20^ Its mortality rate increases with the stage of sepsis, ranging from 12% to 60%.^21^ The absence of an early recognition and a prompt management of patients with sepsis can develop into more severe stages and even death.^22,23^

In the last few years, and in order to deal with this situation, guidelines and campaigns have appeared pointing to the convenience of early recognition and proper management of sepsis.^7–10,23^ One of these is the Surviving Sepsis Campaign that developed guidelines for management of severe sepsis and septic shock in 2004 and updated in 2008 and 2013.^8–10^ As a result, many hospitals and studies implemented these bundles and reported benefits such as the decrease in the progression of organ failure and the mortality rate. However these protocols are used to guide resuscitation of severe sepsis or septic shock in ED and ICU, not including physicians outside these units and the nursing staff. In our study we found that 81.1% of the patients with sepsis criteria were admitted in non-ICU at the time of the interview. Similar data was found by Esteban et al in a study performed in 3 different hospitals in Madrid, Spain, where only 12% of the patients with sepsis criteria were admitted in ICU.^24^ This data reflects the importance to reinforce sepsis systems alerts outside ICU and ED in order to help HCPs in early recognition and proper management of sepsis.

The role of the CM or ID specialist in the early warning of sepsis is limited because it is confined to the identification of microorganisms and their antimicrobial sensitivity which is frequently impossible and always delayed. Our study shows that BCs requested to a Microbiology Department may be a good, complementary source of sepsis alert and an excellent watchdog for the overall hospital sepsis surveillance system. Our study also shows that the telephone interview is welcome and the recommendations made generally implemented.

To our surprise, we found that 40% of patients in group A did not have criteria of sepsis but this was also an excellent source for intervention in favor of a better antimicrobial stewardship. In our opinion, BCs should not be ordered for patients with isolated fever, without a prior physical examination. We observed that the identification of sepsis as a clinical condition is poor. Less than half of HCPs identified correctly a case of sepsis in patients whose BCs had just been sent to the Microbiology Department. The best scores of recognition of sepsis were found in intensive care specialists. Other studies published similar data, showing a lack of knowledge about sepsis among other physicians.^25,26^ Unlike our study, in which we evaluated the HCPs knowledge at the same moment of the evaluation of the patient, these studies only analyzed theoretical knowledge about sepsis.

We found that, in most cases and, at the moment of the extraction of BCs and upon suspicion of sepsis, lactic acid, and procalcitonin were scarcely requested. Different studies have shown that these are good markers in sepsis management and outcome^27–29^ and purportedly a good source for sepsis alert.

In our study, the recommendations given by the CM related to diagnostic testing and antibiotic treatment were highly followed. Other authors that observed the impact of recommendations from ID or CM specialists to other physicians showed a high adherence to the recommendations improving prognosis, reducing antibiotic treatment and decreasing mortality in patients with infectious diseases.^30–35^ However telephone calls had not been deeply studied.^36,37^ Forsblom et al^37^ investigated the impact of informal ID specialist's consultation versus telephone consultation in the management of *S. aureus* bacteremia and found that telephone consultations were inferior than bedside consultations. Our study shows however, that bedside consultations would probably be impossible in most institutions for every single patient from whom BCs are sent to the Microbiology Department. In the present study, to better define the impact of a telephone call from CM in the alert of sepsis, we performed a protocolized interview and issued recommendations.

Sepsis also involves a large financial burden and a high consumption of resources. In 2008, the United States of America spent 14.6 billion dollars on sepsis treatment.^38^ Estimating that each septic patient consumes about 22,000 to 26,000 dollars.^1^ Our study did not intend to make a detailed economic impact of the intervention but we could demonstrate a clear cut cost-effectiveness of a telephone call just by considering the expenses in acquisition of antimicrobials.

Among the limitations of our study, we have to consider that this is a single-center study and our results may not necessarily be extrapolated to other populations. Our institution however has all the characteristics of a large teaching hospital and a long tradition in Clinical Microbiology and Infectious Diseases interventions. It should be noted that we excluded the pediatric population and our results cannot be assumed to work in patients <18 years of age. The satisfaction of the physicians with our interview might have been overestimated because those physicians that did not want to perform the interview were not included in the final score.

On the basis of this study, we consider that the use of a telephone call from the Microbiology Service in patients whose blood cultures were sent to the Department might strongly contribute to an early alert together with a better management of sepsis in the hospital and it is a considerable way to improve antimicrobial stewardship and to reduce antimicrobial expenses.
